# Impact of drought stress on biochemical and molecular responses in lavender (*Lavandula angustifolia* Mill.): effects on essential oil composition and antibacterial activity

**DOI:** 10.3389/fpls.2025.1506660

**Published:** 2025-04-09

**Authors:** Ramin Hosseini, Mahsa Heidari

**Affiliations:** ^1^ Biotechnology Department, Faculty of Agriculture and Natural Resources, Imam Khomeini International University, Qazvin, Iran; ^2^ Forestry and Forest Tree Breeding Department, Georg-August University, Göttingen, Germany

**Keywords:** antioxidant, drought stress, essential oil, gene expression, lavender

## Abstract

Drought stress significantly influences the physiological, biochemical, and molecular processes in plants, directly impacting their growth and defense mechanisms. This study evaluates the response of *Lavandula angustifolia* (lavender) to different levels of water deficit, with field capacity (FC) treatments set at 20%, 40%, 60%, and 80%. We assessed various biochemical parameters, including protein content, chlorophyll a and b levels, flavonoid and phenolic content, and antioxidant activity, to determine how drought stress affects lavender’s primary and secondary metabolism. As water availability decreased, we observed a reduction in total protein and chlorophyll content, while the highest levels of flavonoids, phenolics, and antioxidant activity were recorded in control plants at 80% FC. Gene expression analysis of key terpene synthase genes revealed differential expression patterns, with linalool synthase and α-pinene synthase peaking at 40% FC, and 1,8-cineole synthase and β-phellandrene synthase reaching their highest activity under severe drought (20% FC). Despite this, a clear correlation between gene expression and metabolite accumulation in essential oils was not observed. Drought-induced changes in essential oil composition were associated with enhanced antibacterial activity, particularly against foodborne Gram-positive and Gram-negative bacteria, suggesting that water stress can modulate the therapeutic potential of lavender oil.

## 1 Introduction

Plants face numerous environmental stresses during their lifecycle, such as high temperatures, salinity, and water deficit, all of which can negatively impact their growth and reduce their overall lifespan (Nareshkumar et al., 2020). However, plants have evolved mechanisms to defend against such harsh environmental conditions through biochemical, physiological, and molecular responses (Mittal et al., 2018; Rao et al., 2019). With the advent of global warming, drought stress has become increasingly prevalent worldwide. One of the major stresses limiting plant productivity in arid and semi-arid regions is water scarcity, which reduces photosynthesis and consequently affects plant growth and yield (Zhang et al., 2006).

Water deficiency in plants can be either temporary or permanent, often arising during the hotter seasons when water potential becomes more negative. In response to drought stress, plants increase the production of specific compounds to minimize damage (Nascimento et al., 2000). This boost in secondary metabolites is linked to the plant’s defense mechanisms against stress (Dunford and Vazquez, 2005). Plants frequently encounter harsh abiotic stresses, leading to various anatomical, physiological, and biochemical changes (Kapoor et al., 2020). One of these changes is the fluctuation in photosynthetic pigments, such as chlorophyll a, chlorophyll b, and total chlorophyll (Szekely-Varga et al., 2020). Efficient photosynthesis is essential for plant growth, yield, productivity, and survival (Zahra et al., 2023), but drought stress often limits this process (Hashem et al., 1998; Wang et al., 2023). Chlorophyll content serves as an indicator of photosynthetic capacity, with drought stress reducing chlorophyll levels, particularly chlorophyll b, leading to an increased chlorophyll a to b ratio and darker leaves. The reduction in chlorophyll content during drought has been linked to increased chlorophyllase enzyme activity (Shin et al., 2021).

Drought stress affects gene expression (; Fontez et al., 2023) as well as the production of volatile and secondary metabolites in medicinal plants (Gonçalves et al., 2023). In lavender, for example, essential oil content and linalool levels increase under drought stress, however, there were differences in drought stress tolerance and the essential oil quality and quantity between the 11 cultivars investigated (Saunier et al., 2022) (). In most plants, the production of monoterpenes is closely linked to transcription. For instance, the accumulation of linalool in *L. angustifolia* is directly related to linalool synthase gene transcription, just as menthofuran production in wild mint is transcriptionally regulated (Mahmoud and Croteau, 2003). The linalool synthase gene responsible for linalool production, a major component of lavender essential oil, is strongly expressed in secretory glands (Liu et al., 2024). Water deficits can cause significant changes in plant metabolism, including the production of secondary metabolites (Soltanipour, 2005). Secondary metabolites are diverse phytochemicals found in plants. They are important substances to protect plants and assist them to adapt to the environmental conditions including salt stress, drought, high temperature and etc. There is a vast difference in the level, kind, quantity and quality of secondary metabolites, depending on the plant species and also in response to stress they may change. Secondary metabolites are classified into three groups: nitrogen-possessing (glucosinolates and alkaloids) compounds, phenolics (flavonoids and phenylpropanoids) and terpenes (Fouad et al., 2023). Phenolic compounds are also vital for plant defense and survival, playing a role in growth regulation, reproduction, and protection from UV rays, insects, and pollution Prior et al., 2005).

Moreover, water stress disrupts the electron transport chain, leading to the production of reactive oxygen species (ROS) (Saraswathi and Paliwal, 2011). Antioxidants are compounds that prevent or delay the oxidation of lipids or other molecules by inhibiting the initiation or extension of oxidative reactions. Plants serve as a major source of natural antioxidants, which include carotenoids, flavonoids, cinnamic acids, benzoic acid, folic acid, ascorbic acid, and tocopherols. These antioxidants neutralize reactive oxygen species (ROS), offering protection against oxidative damage (Bouayed et al., 2007). Flavonoids, specifically, act as antioxidants, enzyme inhibitors, and protectors against UV light (Harborne and Williams, 2000; Yang et al., 2022). Lavender essential oil exhibits a wide range of biological activities, including antioxidant (Danh et al., 2013), antibacterial, and anti-inflammatory effects (Giovannini et al., 2016), making it a promising subject for further research.

Lavender, a member of the Lamiaceae family, is a small, highly branched perennial bush resistant to water shortages. Its leaves and flowers contain over 40 different compounds in their essential oils, with linalool acetate, linalool, cineole, camphor, and geraniol being the most prominent 6) (Caprari et al., 2021). Among the numerous essential oil-producing plants, *Lavandula angustifolia* Mill. stands out as one of the most valuable (Crisan et al., 2023). Lavender essential oil is widely used as a flavoring agent in various food products, such as chewing gum, candies, and beverages, and it also acts as a strong antimicrobial agent, helping to prevent food spoilage (Crisan et al., 2023).

This study aims to explore the effects of drought stress on lavender and to unravel the molecular mechanisms underlying its response to water scarcity. Understanding these mechanisms is crucial for identifying how plants minimize damage from oxidative stress and enhance their drought resistance through changes in biochemical and physiological processes.

## 2 Materials and methods

### 2.1 Drought stress treatments

To conduct this experiment, rooted cuttings of lavender plants were placed in plastic pots filled with soil, measuring 24 cm in height and 22 cm in aperture diameter. Four levels of field capacity (FC) water treatments were applied: 80% (control), 60% (low drought stress), 40% (moderate drought stress), and 20% (severe drought stress). The experiment was arranged in a completely randomized design with three replicates per treatment and conducted in a greenhouse. First, a 100 cc sample of saturated soil was collected using a sampling ring, weighed, and then dried in an oven at 105°C for 24 h. The dry weight of the soil and the ring was measured, and the FC was calculated using the formula:


Moisture in 100 cc of soil=(the weight of the rim and wet soil−the weight of the rim and dry soil)

This value was multiplied by the volume of soil in the pot (approximately 8 L) to determine the total moisture in the pot, which was calculated as 2.56 L, representing 100% FC. Based on this, irrigation amounts for the different treatments were adjusted to 80%, 60%, 40%, and 20% FC by applying 2, 1.5, 1, and 0.5 L of water, respectively, every three days over the course of three months. Soil moisture was regularly monitored using a moisture meter calibrated for the specific soil used in the study. Leaf samples from each treatment group were collected, immediately frozen in liquid nitrogen to prevent RNase activation and RNA degradation, and stored at -80°C for later analysis.

### 2.2 Protein extraction and measurement

Protein content in the plant samples was determined using a standard curve prepared with bovine serum albumin. Absorbance was measured in a spectrophotometre (Labomed UV-3200, UK) at 595 nm wavelength, using a 1 ml glass cuvette. Total protein was measured according to the method (Bradford, 1976). The Bradford assay method is actually based on the equilibrium between three forms of Coomassie Blue, which turns blue when bound to protein. Bradford solution is red in highly acidic conditions and has two protons, and its maximum absorption is at 470 nm, which loses two protons when combined with protein and turns blue. Solutions of bovine serum albumin protein (BAP) were prepared with 50, 100, 150, 200, 250, 300, 350, 400, 450 and 500 µg/µl concentrations and a standard curve was prepared using these concentrations of BAP. Twenty microliters of standard samples were mixed with 980 microliters of Bradford and a standard curve was drawn. In order to measure the protein concentration of unknown samples, 20 µl of the sample were mixed with 980 µl of Bradford and after 5 min, the reaction mixture was transferred into a 1 ml glass cuvette and the absorbance was read at 595 nm. The protein concentration was reported in µg/ml (Bradford, 1976; Sapan and Lundblad, 2015).

### 2.3 Chlorophyll extraction and measurement

A 200 µl aliquot of powdered leaf tissue was combined with 800 µl of 100% acetone, gently vortexed, and then stored at -20°C for 1 h. Chlorophyll content was measured by recording absorbance at 645 nm and 663 nm, using pure acetone as a blank using a spectrophotometre (Labomed UV-3200, UK). Chlorophyll concentrations in all samples were calculated in triplicate, and the results were expressed in µg/ml using the formulas provided by Arnon (1949)


Chlorophyll a=(12.7×B)–(2.69×A)

Chlorophyll b=(22.9×A)−(4.68×B)

Total chlorophyll=(20.2×A)+(8.02×B)

A= Absorbance at 663 nm and B= Absorbance at 645nm

### 2.4 RNA extraction

Immediately after sampling, the plant tissues were frozen in liquid nitrogen and transported to the laboratory. The samples were ground into a fine powder using a pestle and mortar that had been double autoclaved, with liquid nitrogen to maintain low temperature. Approximately 50 mg of the powdered tissue was transferred into a sterile 2 ml microtube. A 200 µl volume of extraction buffer (100 mM Tris-HCl pH 8.0, 10 mM EDTA, 0.1 M LiCl, 1% w/v SDS) was added to the powder, followed by rapid vortexing for 1 min. The samples were then incubated at room temperature for 10 min. The aqueous phase was transferred into a new tube and an equal volume of Chl: Iaa (24:1, v/v) was added, shaken, and vortexed until the two phases form an emulsion; Subsequently, the mixture was centrifuged at 12,000 rpm for 15 min at 4°C. The aqueous phase was collected and 1/3 volume ice-cold 8 M LiCl was added. Tubes were inverted gently and placed at 80 °C for 30 min. Then tubes were allowed to thaw and centrifuged at 12000 rpm for 30 min. The supernatant was taken gently by a sampler and poured out. The pellet was dissolved in 200 µL RNase-free water, and then 0.1 volume 3M sodium acetate (pH 5.2) and 2 volumes ice-cold absolute ethanol were added. Tubes were inverted gently and placed at80 °C for 30 min. The RNA pellet was collected by centrifugation at 12000 rpm for 30 min and then washed by 70% (v/v) ice cold ethanol, air dried, and dissolved in 20–30 µL RNase-free water and stored at 80 °C (Sabzevari and Hosseini, 2014). The concentration of the extracted RNA was determined using a NanoDrop spectrophotometre (model VVD3200, Lebomed, Germany). The quality of the RNA samples was assessed by running them on a 1.5% (w/v) agarose gel, followed by visualization and photography using a UV trans-illuminator (UVP, USA). All pipette tips and microtubes were autoclaved twice to prevent RNase contamination.

### 2.5 cDNA synthesis

cDNA synthesis was performed using the RevertAid M-MuLV Reverse Transcriptase Kit (CinaClon, Tehran, Iran). To design nucleotide primers for the qRT-PCR reaction, gene sequences were first retrieved from the NCBI database. Primer design (**Supplementary Table 1**) was conducted using Oligo 5, v3.0 software, and the specificity of the primers, including the absence of non-specific binding and primer-dimer formation, was confirmed using Primer Blast from the NCBI database.

The materials used for the qRT-PCR reaction are listed in **Supplementary Table 2**, with a final RNA concentration of 1 μg. Oligo dT and RNA were added to the reaction tube and the final volume was adjusted to 12.5 μl using nuclease-free deionized water. The mixture was incubated at 65°C for 5 min, followed by immediate cooling on ice for 5 min. The remaining steps for cDNA synthesis were performed according to the manufacturer’s instructions.

### 2.6 qRT-PCR reaction

The expression levels of the genes Linalool Synthase (accession number DQ263741.1), β-phellandrene Synthase (accession number HQ404305.1), 1,8-Cineole Synthase (accession number JN701461.1), and α-pinene Synthase were assessed using qRT-PCR. 18S rRNA (accession number LC373552.1) served as the internal control. All reactions were conducted using Cyber Green reagent (Aminsan, Iran) in a total volume of 20 μl, with 40 amplification cycles. The qRT-PCR protocol included an initial denaturation at 94°C for 3 min, followed by 40 cycles of 94°C for 30 s, annealing at 52-54°C (depending on the primer), extension at 72°C for 20 s, and a final extension at 72°C for 5 min.

### 2.7 Preparation of methanolic extract

The methanolic extracts were prepared from the aerial parts of the plant samples using 80% methanol. The plant material was dried at room temperature, ground into a fine powder using a pestle and mortar, and 2 g of the powder was mixed with 20 ml of 80% methanol in a shaker incubator. The mixture was then filtered through Whatman No. 1 filter paper and centrifuged at 6,000 rpm for 5 min. The clear supernatant was collected and transferred to a volumetric flask connected to a rotary evaporator. The extract was concentrated under reduced pressure, then further dried using airflow until it became a viscous, oily substance. The final extract (4.0 mg/ml in mathanol) was stored in tightly sealed containers at -20°C.

### 2.8 Total phenol measurement

The total phenolic content of the plant extracts was determined using the Folin-Ciocalteu method (Pourmorad et al., 2006). A 0.5 ml aliquot of each plant extract or Gallic acid (used as the standard) was mixed with 2.5 ml of Folin-Ciocalteu reagent. Then, 2 ml of 7.5% Na_2_CO_3_ was added to the mixture. The samples were kept in the dark at room temperature for 2 h. A standard curve was created using 1, 10, 25, 50, 100 and 250 µg/ml of Gallic acid and their absorbance was measured at 740 nm. The total phenolic content was measured at 740 nm using a spectrophotometre (Labomed UV-3200, UK) with reference to the standard curve. Results were expressed as milligrams of Gallic acid equivalents (GAE) per gram of dry weight, with four replicates analyzed for each sample.

### 2.9 Total flavonoid measurement

The total flavonoid content was measured using the aluminum chloride colorimetric method (Pourmorad et al., 2006). A 0.5 ml portion of each plant extract was mixed with 1.5 ml of methanol, 0.1 ml of 10% aluminum chloride, 0.1 ml of 1 M potassium acetate, and 2.8 ml of distilled water. The mixture was allowed to stand at room temperature for 30 min. A standard curve was created using 1, 10, 25, 50 and 100 µg/ml of quercetin. Absorbance was measured at 415 nm using a spectrophotometre (Labomed UV-3200, UK). The total flavonoid content was expressed as milligrams of quercetin equivalents (QE) per gram of dry weight, with each sample analyzed in four replicates.

### 2.10 Measurement of antioxidant activity

The antioxidant activity of the plant extracts was evaluated using the DPPH (1,1-diphenyl-2-picrylhydrazyl) free radical scavenging assay, following the method of Stojičević et al. (2008). Plant extracts were prepared at concentrations of 0.5, 1, 2, and 4 mg/ml. A 0.3 ml aliquot of each extract was mixed with 2.7 ml of 0.01 mg/ml DPPH methanolic solution, vigorously stirred, and incubated in the dark at room temperature for 30 minutes. Absorbance was measured at 517 nm using a spectrophotometre (Labomed UV-3200, UK) to obtain the sample absorbance (As). A solution of DPPH in methanol served as the control (Ac), while methanol mixed with the plant extract served as the blank (Ab). Each sample was tested in triplicate. The percentage of DPPH radical scavenging activity was calculated using the following formula:


DPPH=100[1−(As−Ab/Ac)]

where As is the absorbance of the plant extract in the DPPH solution, Ac is the absorbance of the control (DPPH solution only), and Ab is the absorbance of the plant extract without DPPH. Gallic acid was used as the reference standard.

### 2.11 Preparation of essential oil

The leaves of the plant were thoroughly dried in a dark, dry environment. Essential oil extraction was performed using hydrodistillation with a Clevenger apparatus. For the extraction process, 100 g of powdered leaves were placed into the Clevenger flask, and approximately 600 ml of distilled water (dH_2_O) was added to soften the plant material. After distillation for 5 h, the essential oil was collected and dehydrated using sodium sulfate. It was then dissolved in dimethyl sulfoxide and stored in dark-colored containers at -20°C.

### 2.12 GC-MS analysis

The extracted essential oil was analyzed using a Shimadzu 2214 gas chromatography (GC) system equipped with a flame ionization detector (FID). The column used was 32 meters in length with a film thickness of 2.24 mm and an internal diameter of 2.24 mm, fitted with a 5-HP micrometer. The initial oven temperature was set at 42°C and held constant for 12 minutes. Afterward, the temperature was increased at a rate of 3°C per min to reach 242°C, and then further increased to 322°C at a gradient of 4°C per min. The injection site was maintained at 242°C, and helium was used as the carrier gas at a flow rate of 1.1 ml/min.

For component identification, the essential oil (1µl) was analyzed on an Agilent 7890A GC system, coupled with an Agilent 5975C mass spectrometer (MS). The ionization source temperature in the MS was set at 232°C, with an ionization voltage of 32 electron volts. The constituents of the essential oil were identified by calculating the retention indices and comparing them with reference values (Adams and Sparkman, 2007), as well as by comparing the mass spectra with data from the internal GC-MS library.

### 2.13 Antibacterial activity

The antibacterial properties of the essential oil were tested against four bacterial strains: *Staphylococcus aureus*, *Escherichia coli*, *Pseudomonas aeruginosa*, and *Bacillus subtilis*. A fresh 24-h culture of each bacterial strain was prepared by inoculating a loopful of culture into 5 ml of Mueller-Hinton broth and incubating at 37°C for 24 h. The bacterial suspensions were then used to inoculate Mueller-Hinton agar plates. To assess the antibacterial activity, sterile paper discs were soaked in the essential oil (20 µl) and placed on the inoculated agar plates, ensuring appropriate spacing between the discs and the plate edges. Discs impregnated with ampicillin and streptomycin (50 μg/ml) served as positive controls. The plates were incubated at 37°C for 24 h. The antibacterial activity was evaluated by measuring the diameter of the inhibition zones around the discs. All experiments were conducted in triplicate.

## 3 Results and discussion

In this study, lavender plants (*Lavandula angustifolia* Mill.) were subjected to four levels of drought stress: 80% (control), 60%, 40%, and 20% field capacity (FC) in a greenhouse environment. Various parameters were examined, including protein and chlorophyll contents, phenolic and flavonoid compounds, antioxidant capacity, essential oil content, antibacterial properties of the essential oil, and essential oil composition across all treatments. Additionally, the expression of four key genes responsible for encoding enzymes involved in essential oil production was analyzed.

### 3.1 Total soluble protein and chlorophyll content

**Figure 1:**
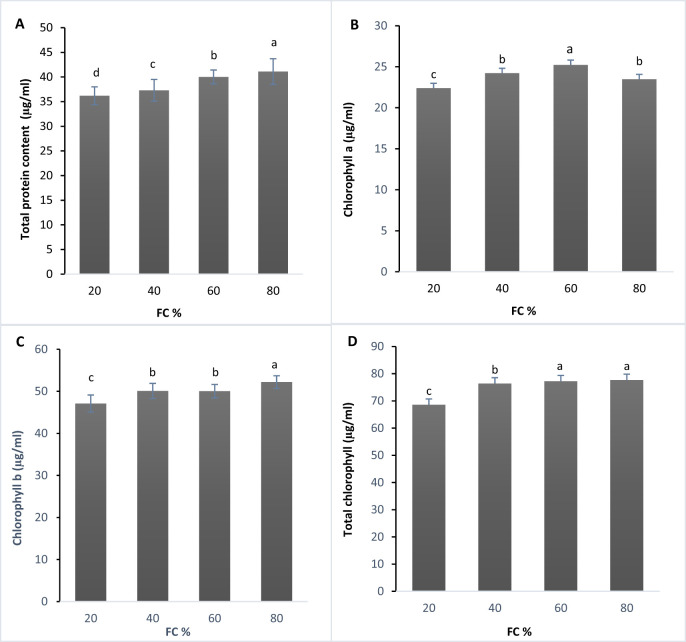
**(A)** Total protein (µg/ml), **(B)** Chlorophyll a content (µg/ml), **(C)** Chlorophyll b content (µg/ml), and **(D)** Total chlorophyll content (µg/ml) in lavender plants under drought stress (20, 40, 60 and 80% field capacity). Different letters indicate significant differences at 1%.

The analysis of variance revealed a significant difference in protein content across treatments at the 1% significance level (**Supplementary Table 3**). The results were categorized into four statistical groups using Duncan’s test. As drought stress increased, total soluble protein content decreased, with the highest protein levels observed at 80% FC and the lowest at 20% FC (**Figure 1A**). Du and Rennenberg (2018) found a similar decline in soluble protein content in *Lavandula* plants under water deficit, though the plants recovered after re-irrigation. The trend of decreasing protein content from 80% to 20% FC aligns with findings from other studies (Du and Rennenberg, 2018; Szekely-Varga et al., 2020), suggesting that ROS-induced protein denaturation might be responsible (Ahluwalia et al., 2021).

Chlorophyll content, a critical factor for photosynthesis, is significantly affected by water stress (Rahdari et al., 2012). The effects of drought stress on chlorophyll a, b, and total chlorophyll content were also significant at the 1% level (**Supplementary Table 3**). Based on Duncan’s test, chlorophyll a, b, and total chlorophyll content were divided into three statistical groups (**Figures 1B–D**).

The lowest chlorophyll a content was recorded at 20% FC, while the highest was observed at 60% FC (**Figure 1B**). For chlorophyll b, the highest level was found at 80% FC, with the lowest at 20% FC (**Figure 1C**). In terms of total chlorophyll content, the highest amount was measured at 80% FC, whereas the lowest was observed at 20% FC (**Figure 1D**).

Although chlorophyll content typically decreases under drought stress, some studies have reported the opposite. For instance, Rolando et al. (2015) found that potato plants under moderate drought stress exhibited an increase in chlorophyll content. Similarly, Raja et al. (2020) observed a decrease of 80% in chlorophyll and 57% in carotenoid content in tomato plants subjected to both drought and heat stress. Chlorophyll content during drought stress is affected by reactive oxygen species like O_2_− and H_2_O_2_, which cause lipid peroxidation, ultimately leading to chlorophyll degradation (Karimpour, 2019).

### 3.2 Total phenolic and flavonoid content

**Figure 2:**
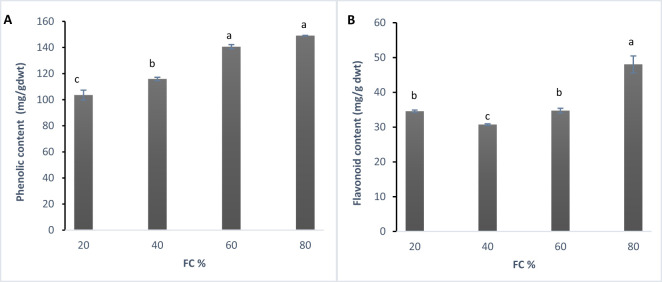
**(A)** Total phenolic content (mg/g dwt) and **(B)** Total flavonoid content (mg/g dwt) in lavender plants under drought stress (20, 40, 60 and 80% field capacity). Different letters indicate significant differences at 1% probability level.

The analysis of variance showed that changes in phenolic content and flavonoids were significant at the 1% probability level (**Table 1**). According to Duncan’s test, the data were divided into four statistical groups. The highest phenolic content was recorded at 60% field capacity (FC), with a decrease in phenolic content as drought stress increased (**Figure 2A**).

**Table 1:** Mean squares and the significance level of flavonoid and phenol contents and antioxidant activity measured by DPPH in lavender plants under drought stress (20%, 40%, 60% and 80% field capacity).

S.V.	Flavonoid	Phenol	DPPH
Treatment	623367.3**	21727.8**	14.95**
C.V.	29.65	33.77	19.61

** means significance at 5 % probability level.

In this study, phenolic content was at its highest at 80% (control) then decreases were observed at 60% (low drought stress), followed by declines at 40% and 20% FC. This decrease in phenolic compounds might be explained by the idea that, upon exposure to drought, plants may experience a shortage of precursors due to reduced biosynthesis or increased degradation. Supporting this observation, Albergaria et al. (2020) reviewed 343 studies on phenolic changes under water deficit. They screened them and eventually 34 articles were selected for the review. They concluded that the common assumption of increased phenolic content in response to drought may not be universally true. In fact, many species show a decrease in phenolic compounds under water stress. However, in contrast to these findings, Shabankareh et al. (2021) reported a significant rise in total phenolic and flavonoid content in three *Lavandula angustifolia* genotypes in response to drought, with the increase accelerating as water deficit intensified and prolonged. The analysis of variance revealed that changes in flavonoid content were significant at the 1% probability level. Duncan’s test results categorized the data into three statistical groups. The highest flavonoid content was observed at two stress levels: 80% and 20% field capacity (FC), while the lowest content was recorded at the 60% and 40% FC stress levels (**Figure 2B**).

Phenolic compounds are widely regarded as indicators of stress tolerance by many researchers (Caliskan et al., 2017; Kumlay et al., 2022). Szekely-Varga et al. (2020) studied the effects of drought and salinity stress on two *Lavandula angustifolia* varieties, reporting that phenolic compounds and flavonoids increased in response to oxidative stress caused by drought. However, this increase was not accompanied by a clear involvement of antioxidant enzymes. Both lavender varieties exhibited a significant rise in total phenolic compounds (TPC) under water stress, with a more pronounced increase in total flavonoids in plants subjected to drought treatments. Phenolic compounds, especially flavonoids, have been shown to play various roles in stressful conditions (Shah and Smith, 2020), particularly as potent antioxidants. By reducing oxidative damage, they help plants enhance tolerance to stress (Sharma et al., 2012; Hussain et al., 2013; Al Hassan et al., 2017). Several authors suggest that plants may have evolved to accumulate phenolic compounds and flavonoids as a key mechanism for coping with stress (Sharma et al., 2017; Rodriguez-Calzada et al., 2019; Arora et al., 2018, Arora et al., 2020).

The data show that the flavonoid content in lavender plants started with the highest level at 80% FC. As drought stress increased to 60% FC, flavonoid level decreased and it continued to 40% FC. This suggests that during moderate drought, plants may reduce flavonoid production and shift their antioxidant defense towards other mechanisms, such as enzymatic antioxidants like superoxide dismutase (SOD), catalase, and peroxidases. However, under severe drought stress (20% FC), flavonoid production spiked again, possibly to mitigate the damage caused by extreme stress. Further research is needed to confirm these findings.

### 3.3 Antioxidant activity measured by DPPH

The analysis of variance for the DPPH (2,2-Diphenyl-1-picrylhydrazyl) assay showed significant differences between samples at the 1% probability level (**Table 1**). The DPPH assay is a reliable, fast, and practical method for measuring the antioxidant capacity of various substances. It uses free radicals to assess a substance’s ability to donate hydrogen or act as a free radical scavenger. Antioxidant activity is also influenced by the structure of phenolic compounds, particularly the number and arrangement of hydroxyl groups (Krol et al., 2014).

The interaction of DPPH with an unpaired electron results in the formation of a purple color, which exhibits strong absorbance at 517 nm (Baliyan et al., 2022). This method is commonly used by researchers to assess antioxidant activity.

**Figure 3:**
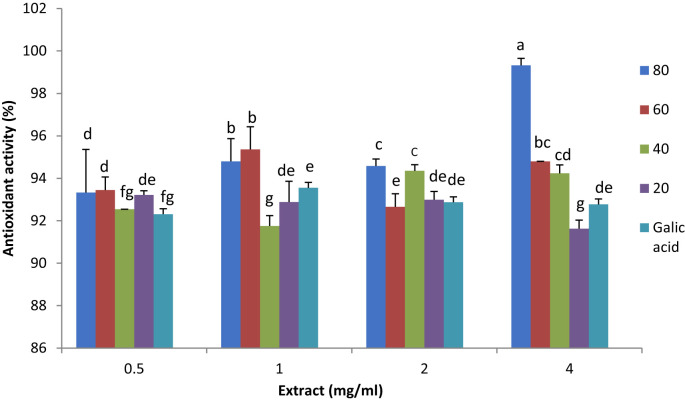
Antioxidant activity and the effect of plant extract concentration (0.5, 1.0, 2.0, 3.0 and 4.0 mg/ml) in lavender plants under drought stress (20%, 40%, 60% and 80% field capacity) measured by DPPH assay. Different letters indicate significant differences at 1% probability level.

As shown in **Figure 3**, various concentrations of plant extracts, ranging from 0.5 to 4 mg/ml, were utilized for the DPPH activity assay to investigate the effect of plant extract concentration. Gallic acid (50 µg/ml) was used as the control. The concentrations effect is not observed as one might expect, i.e. by increasing the concentration of plant extract, the antioxidant capacity does not show considerable increases. It can be explained by the saturation of DPPH assay, meaning that all or most of the oxidative molecules have been scavenged in lower plant extract concentrations, or in higher plant extract concentration antioxidant molecules aggregate and do not show the expected activity.

In 1 mg/ml plant extract, the extract from 60% FC plants showed the highest antioxidant activity, and the lowest activity was observed in 40% FC plant extract. It was the same for 1 ml plant extract, however, in 2 ml plant extract the highest antioxidant activity was observed in 80 and 40% FC plant extracts. In 4 ml plant extract, 80% FC plant extract depicted the highest activity and it decreased to 20% FC. (**Figure 3**). This decrease may be due to the reduction or degradation of precursors for non-enzymatic antioxidant compounds as stress continued. A similar decline in DPPH activity was reported by Kowalczewski et al. (2020), who also noted that the method used to determine antioxidant activity and plant genotype can influence the assay results. The effect of genotype on antioxidant capacity was further supported by Shabankareh et al. (2021), who investigated the total antioxidant activity of three *Lavandula angustifolia* genotypes (H, S, and M) under water deficit. They found that genotypes responded differently to drought, with H and S genotypes showing increased antioxidant activity, though the increase was not as pronounced in the M genotype.

### 3.4 Gene expression analysis, essential oil content and composition

Gene expression assays were conducted using qRT-PCR with three technical and experimental replications for each sample. SYBRgreen was used for detection, and reactions were performed in 70-well microplates with a final volume of 20 μl. All genes depicted significant differences under drought stress (**Table 2**).

**Table 2:** Mean squares and significance level in the expression of linallol synthase, 1,8-lineol synthase, α-Pinene synthase β-Phellandrene synthase involved in EO biosynthesis in lavender plants under drought stress (20%, 40%, 60% and 80% field capacity).

S.V.	Linalool synthase	1,8- lineol synthase	α-Pinene synthase	β-Phellandrene synthase
Treatment	26.54**	102.71**	188.09**	13.65**
C.V.	17.33	15.85	22.5	14.78

** means significance at 5 % probability level.

**Figure 4:**
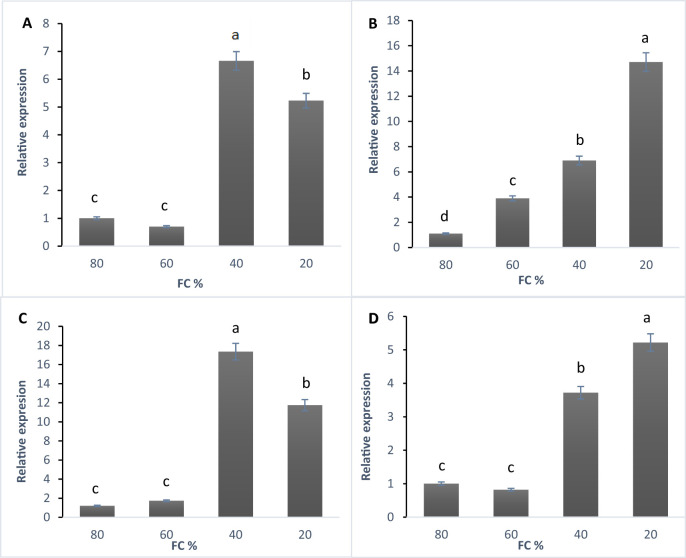
**(A)** The expression of linalool synthase gene, **(B)** 1,8- cineol synthase gene, **(C)** α-Pinene synthase gene, and **(D)** β-Phellandrene synthase gene in lavender plants under drought stress (20%, 40%, 60% and 80% field capacity) revealed by qRT-PCR. Different letters indicate significant differences at 1% probability level.

According to the qRT-PCR data, the linalool synthase gene was expressed at all stress levels (**Figure 4A**). The highest expression level occurred at 40% FC, with a sixfold increase compared to the control. At 20% FC, expression increased fivefold. The lowest expression was seen at 60% FC, showing a reduction compared to the control (**Figure 4A**). The expression of the 1,8-cineole synthase gene increased as drought stress intensified, peaking at 20% FC with a 14-fold rise compared to the control. The lowest expression was at 60% FC, though it was still four times higher than the control (**Figure 4B**). The α-pinene synthase gene was expressed at all stress levels, with the highest expression at 40% FC, where it increased 17 times compared to the control. At 20% FC, expression increased 11 times, while the lowest expression occurred at 60% FC, with a slight rise compared to the control (**Figure 4C**). Expression of the β-phellandrene synthase gene also increased under drought stress, reaching its highest level at 20% FC with a fivefold increase over the control. At 40% FC, expression increased 3.5 times, while 60% FC showed the lowest expression, with a decrease relative to the control (**Figure 4D**).

The accumulation of lavender essential oils is influenced by various factors, including the timing of release, developmental stage, light, temperature (Seira et al, 2023), and environmental conditions such as water deficit, as demonstrated in this study. Seira et al. (2023) used quantitative qRT-PCR to monitor the relative expression of linalool synthase (LaLINS), limonene synthase (LaLIMS), and terpene synthase-like (LaTPS-l) genes in the flowers of *L. angustifolia* cv. Etherio at stages 3 to 5, every three hs over two consecutive days. Their findings revealed a rhythmic pattern of gene expression, with the highest levels occurring around midday to 3:00 PM. In particular, LaLINS expression increased threefold before rapidly declining in the evening. This time-dependent release of volatile compounds likely serves to attract pollinators and repel predators, optimizing resource use efficiency (Fenske and Imaizumi, 2016; Zeng et al., 2017). Demissie et al (2011), Demissie et al, 2012) investigated the expression of several genes involved in essential oil production in the leaves and flowers of *L. angustifolia*, including β-phellandrene synthase (LabPHLS), terpene synthase-like (LaTPS-I), linalool synthase (LaLINS), and limonene synthase (LaLIMS). Their study found that the composition and concentration of essential oils were not directly linked to the expression of the genes responsible for their synthesis. For example, β-phellandrene accumulates in large amounts in *L. angustifolia* leaves, while linalool is predominantly produced in the flowers. Despite this, microarray data showed no significant differences in the expression of linalool synthase between leaves and flowers, a pattern also observed with LaTPS-I (Demissie et al., 2011). However, in some cases, monoterpene accumulation has been shown to correlate with the transcript levels of corresponding monoterpene synthase genes. For instance, Lane et al. (2010) reported a direct correlation between linalool accumulation and the transcript levels of the linalool synthase gene in *L. angustifolia*. Similarly, Mahmoud and Croteau (2003) found that the production of menthofuran correlated with the expression of its respective gene.

Monoterpene synthases often produce multiple products from the same substrate. For example, Bohlmann et al. (1999) demonstrated that β-phellandrene synthases from grand fir and tomato yielded a variety of products, including 52% β-phellandrene, 34% β-pinene, 8.5% α-pinene, and 6% 4S-limonene. Another critical factor affecting terpene composition is substrate availability. Schilmiller et al. (2009) reported that when neryl diphosphate (NPP) was used as a substrate for tomato β-phellandrene synthase, β-phellandrene was the primary product, with small amounts of carene, α-phellandrene, γ-terpinene, and limonene. However, when geranyl diphosphate (GPP) was used, the enzyme produced β-phellandrene, myrcene, and ocimene in similar quantities, along with trace amounts of linalool.

### 3.5 Essential oil content

**Figure 5:**
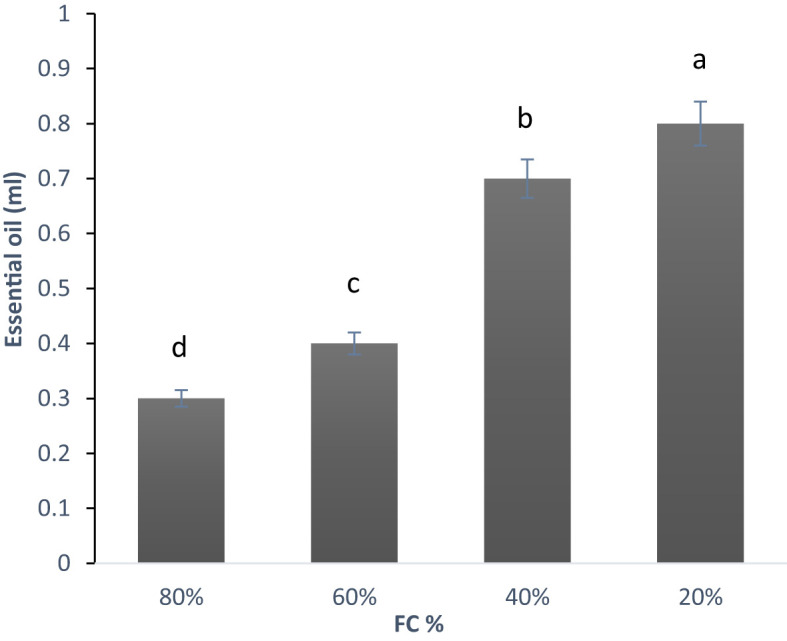
Changes in the essential oil content in lavender plants under drought stress (20%, 40%, 60% and 80% field capacity). Different letters indicate significant differences at 1% probability level.

The analysis of variance for samples under drought stress showed that essential oil content was significantly affected at 1% probability level (**Supplementary Table 4**). As shown in the diagram (**Figure 5**), the essential oil content increased as the level of drought stress intensified (**Table 3**).

**Table 3:** The main components detected by GC-MS in the essential oil in lavender plants under drought stress (20%, 40%, 60% and 80% field capacity).

Treatment	α-Cadinol	Borneol	Camphor	1,8-Cineol	β-Pinene	α-Pinene
20%	3.14 c	20.13 a	14.34 c	38.39 ab	3.33 b	2.40 b
40%	3.66 a	15.48 c	17.90 b	36.64 b	3.76 a	2.89 a
60%	3.39 b	18.06 b	14.46 c	40.35 a	2.96 c	2.13 c
80%	2.06 d	17.88 b	18.76 a	41.28 a	1.77 d	1.57 d

Different letters indicate significant differences at 1% probability level.

The lowest essential oil percentage was recorded at 80% FC (0.3 ml), while the highest was at 20% FC (0.80 ml), more than double the amount at 80% FC. As noted by several researchers, drought stress significantly affects both the content and composition of essential oils. For example, Shabankareh et al. (2021) investigated the effects of drought stress on the essential oil percentage and composition in three *Lavandula angustifolia* genotypes. Their findings revealed that drought stress significantly altered both the quantity and makeup of the essential oil, with the impact being genotype-dependent. In two genotypes, the essential oil content increased up to a certain level of stress, followed by a decline. However, in the third genotype, the content continued to rise without reaching a plateau. Additionally, it appears that essential oil content and composition can vary based on geographical location.

The essential oil content and composition in *L. angustifolia* also vary depending on the plant organ used for extraction. Significant variations in essential oil content have been reported in *L. angustifolia* (Giannoulis et al., 2020). Raina and Negi (2012) found that in *L. angustifolia* plants grown in British Columbia, Canada, the essential oil extracted from leaves ranged from 0.7 to 2.9 mg/g fresh weight, while in flowers, it ranged from 13.9 to 15.3 mg/g fresh weight. Several other factors, such as genotype, soil, and climate changes, also influence essential oil variation. Giannoulis et al. (2020) studied the effects of year, genotype, and a bio-stimulant on essential oil percentages in three *L. angustifolia* cultivars during 2018 and 2019, with oil percentages ranging from 1.94 to 2.58%. They observed significant differences across all factors studied, with the bio-stimulant having a positive impact on essential oil production. Similarly, Détár et al. (2020) examined *L. angustifolia* cultivars and the harvest year, reporting essential oil percentages ranging from 1.9–5.2% in 2017 and 2.1–8.2% in 2018, with more pronounced differences between cultivars.

In the present study, the essential oil was extracted from leaves, with the dominant components being 1,8-cineole (38.4%) and borneol (17.83%), along with several minor components (**Supplementarys Table 5** and **Supplementarys Table 6**). Amongst different classes of terpenes, oxygenated monoterpenes (more than 80%) depicted the highest percentage and then in an order, monoterpene hydrocarbons, oxygenated sesquiterpenes and sesquiterpene hydrocarbons contained the rest of the EO composition (**Supplementary Table 7**). The changes in EO composition in lavender under various drought stress levels can arise from more energy consumption by the plant for water absorption, change in protoplast constituents and its osmotic pressure, changes in respiratory pathways and pentose phosphate pathway, and consequently causing impairments in the production of enzymes involved in the biosynthesis of EO (Khorasaninejad et al., 2015).

The essential oil of *L. angustifolia* contains various compounds. Floral oil is primarily composed of linalool, linalyl acetate, camphor, and 1,8-cineole, whereas leaf oil mainly consists of borneol, camphor, and 1,8-cineole (Cavanagh and Wilkinson, 2002; Prusinowska and Smigielski, 2014). Factors such as season, environmental conditions, genotype, and plant nutrition significantly influence the chemical composition of lavender essential oil (Hui et al., 2010). Falk et al. (2009) found that the major components in floral oil were linalool and linalyl acetate, while leaf oil contained predominantly phellandrenes, along with smaller quantities of camphor and borneol.

This aligns with the findings of Hassanpouraghdam et al. (2011), who identified linalool (33.7%), 1,8-cineole (17.1%), borneol (14.7%), and camphor (7.8%) as the main constituents of essential oil extracted from inflorescences. In contrast, the leaf essential oil of *L. angustifolia* showed the highest concentrations of 1,8-cineole (31.9%), borneol (24.0%), and camphor (16.1%).

The response of lavender plants to drought stress and subsequent recovery was examined by Kumlay et al. (2022), who reported that eucalyptol, camphor, and borneol were the primary components of the essential oil. They noted that the percentage of camphor increased with both single and repeated drought stress but declined during recovery and full irrigation. Similar findings were reported by Chrysargyris et al. (2016) and Kulak (2020).

### 3.6 Antibacterial properties

**Figure 6:**
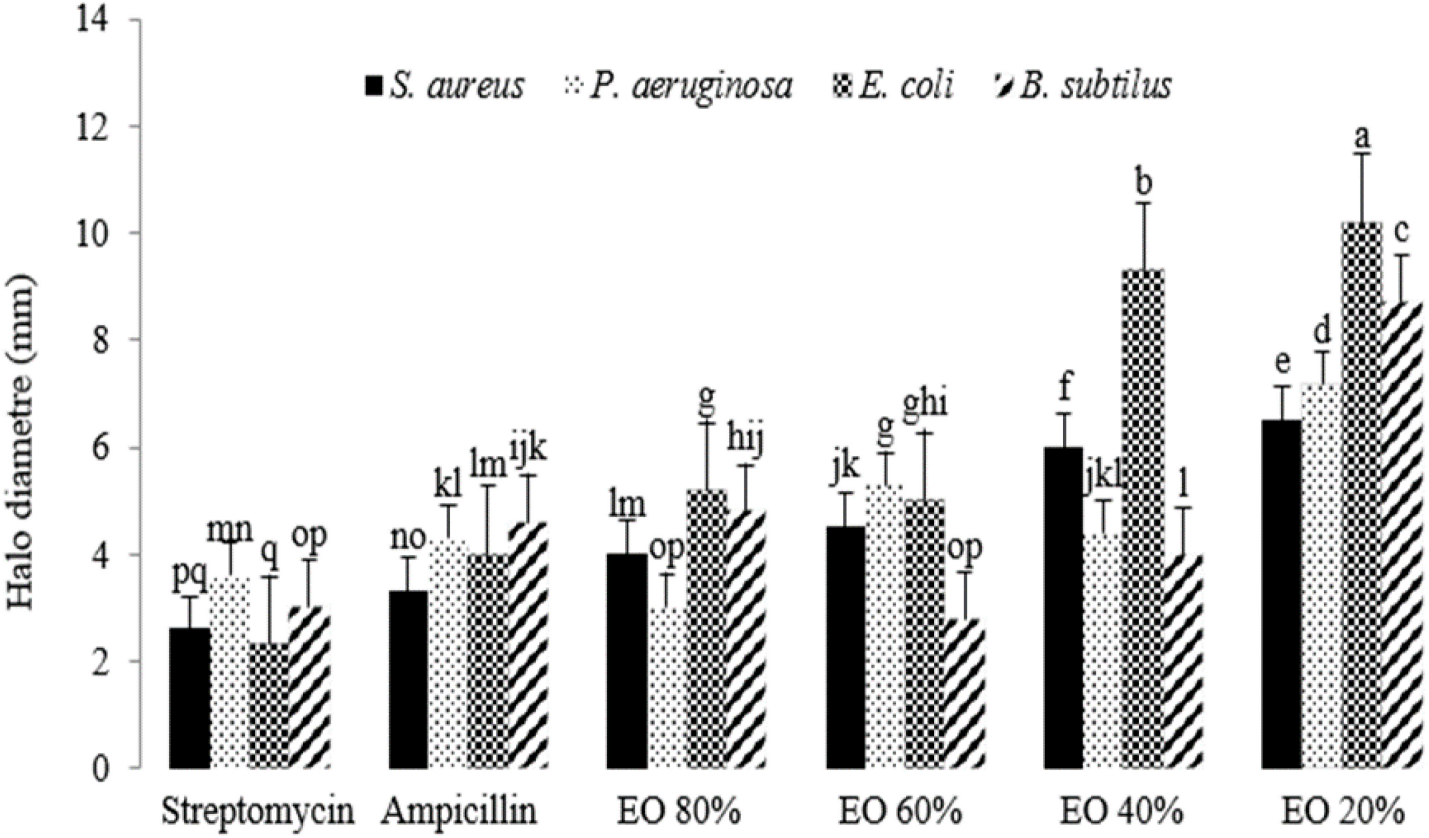
The antibacterial activity of essential oils extracted from lavender plants under drought stress (20%, 40%, 60% and 80% field capacity). Different letters indicate significant differences at 1% probability level.

The antibacterial activity of the essential oil was significant at the 1% probability level (**Supplementary Table 8**). As the intensity of stress increased, the antibacterial properties of the extracted essential oils enhanced compared to the controls, streptomycin and ampicillin. The most significant antibacterial activity was observed with essential oils extracted from plants grown under 40% and 20% field capacity (FC) when tested against *E. coli*. Following *E. coli*, the essential oils exhibited the second highest antibacterial effect against *B. subtilis*. In contrast, the essential oils extracted from plants grown at 80% and 60% FC showed minimal differences in their antibacterial activity. Overall, stress appeared to positively influence the antibacterial properties of the essential oils (**Figure 6**).

There have been significant efforts to discover novel natural compounds with antibacterial properties that are cost-effective and low in toxicity (Guimarães et al., 2019), particularly for use in the food industry (Sonker et al., 2015). This is crucial, as chemical additives used as preservatives in food may lead to various cancers in human tissues (Guimarães et al., 2019). EOs have been utilized as substitutes for chemical preservatives. Guimarães et al. (2019) assessed several components present in the EOs of different plants against *Bacillus cereus*, *Salmonella Typhimurium*, *Escherichia coli*, and *Staphylococcus aureus*. They found that compounds containing hydroxyl groups, such as phenolics and alcohols, exhibited stronger antibacterial activities compared to those composed of hydrocarbons, even in comparison to sulfanilamide. Scanning electron microscopy images indicated that cell death resulting from EO treatment was due to the loss of cell membrane integrity. This finding is corroborated by Merghni et al. (2023), who observed that exposing *S. aureus* cells to various doses of 1,8-cineole led to the accumulation of reactive oxygen species (ROS), followed by cell death. The disruption of ATP synthesis machinery in bacteria has also been cited as another target for EOs (Gill and Holley, 2004).

It has been proposed that the minor compounds present in EOs enhance their antibacterial activity through a synergistic mechanism with the primary compounds (Falleh et al., 2020). The tested bacterial strains included *S. aureus*, *E. coli*, *Pseudomonas aeruginosa*, and *Bacillus subtilis*, all of which can pose risks to human health and cause food poisoning.

The antibacterial activity of lavender essential oil was assessed against four bacteria related to rhinitis: *S. aureus*, *Micrococcus ascoformans*, *Proteus vulgaris*, and *Escherichia coli*. All tested bacteria were affected by the essential oil, with bacterial growth inhibited as the concentration of the oil increased (Hui et al., 2010). Notably, at a concentration of 51 mg/ml, lavender essential oil inhibited the growth of all four bacteria. The antibacterial effect was attributed to the disruption of the cell membrane’s permeability barrier, along with a likely simultaneous loss of chemiosmotic control, contributing to the lethal action of the essential oil. Additionally, lavender essential oil demonstrated significant antibacterial activity, suggesting its potential as a natural treatment for rhinitis patients (Hui et al., 2010).

## 4 Conclusion

This study explored the impact of drought stress on lavender, revealing various physiological and biochemical changes in the treated plants. Both chlorophyll content and soluble proteins decreased under stress, while flavonoid and phenolic contents significantly declined with increasing water deficit. Typically, phenolics and flavonoids play a role in helping plants tolerate abiotic stresses. Although lavender is known for its drought tolerance, the observed decreases in these compounds indicate that its tolerance may be attributed to other antioxidant mechanisms present in the plant. Gene expression related to terpene synthesis increased, and essential oil content rose with higher water stress intensity. Water stress significantly altered both the quality and quantity of essential oil components. The lavender essential oil exhibited strong antibacterial activity against four bacterial strains, with heightened activity correlated with increased drought stress. This increase may result from a rise in the concentration of antibacterial components in the essential oil, influenced by varying levels of drought stress, or it could be due to the enhanced synergistic effects of certain components. Overall, lavender plants demonstrated changes in the quantity and composition of their essential oil, which are critical for the food, pharmaceutical, and medicinal industries. This manipulation allows for adjustments to the quantity and composition of lavender essential oil based on specific requirements by inducing drought stress in the plants.

## Data Availability

The datasets presented in this study can be found in online repositories. The names of the repository/repositories and accession number(s) can be found in the article/**Supplementary Material.**

## Supplementary material

Supplementary Table 1 Primers for the expression analysis of 4 genes involved in EO biosynthesis used for qRT-PCR.

Supplementary Table 2 Components used for gene expression analysis, using qRT-PCR

Supplementary Table 3 The ANOVA on the effect of treatment (stress levels) on total protein and chlorophyll contents in lavender plants under drought stress (20%, 40%, 60% and 80% field capacity) revealed by qRT-PCR

Supplementary Table 4 The ANOVA on the EO content in lavender plants under different levels of drought stress (20%, 40%, 60% and 80% field capacity).** indicate significant at 1% probability level

Supplementary Table 5 Comparison between the components and their quantities in this study with the findings reported by Hassanpouraghdam et al. (2011).

Supplementary Table 6 ANOVA result on the main compositions of essential oils in lavender plants under drought stress (20%, 40%, 60% and 80% field capacity). ** indicate significant at 1% probability level

Supplementary Table 7 The percentage of monoterpene hydrocarbons, oxygenated sesquiterpenes, sesquiterpene hydrocarbons and oxygenated sesquiterpenes in the EO of lavender plants under different levels of drought stress (20%, 40%, 60% and 80% field capacity).

Supplementary Table 8 ANOVA result on the antibacterial activity of essential oils of plants under drought stress (20%, 40%, 60% and 80% field capacity).** indicate significant at 1% probability level
