# Supplementary material for: Impact of drought stress on biochemical and molecular responses in lavender (Lavandula angustifolia Mill.): effects on essential oil composition and antibacterial activity
Source: Front Plant Sci. 2025 Apr 9;16:1506660. doi: 10.3389/fpls.2025.1506660 (PMC12014543; doi:10.3389/fpls.2025.1506660)
Supplement: Supplementary Table 1 — Primers for the expression analysis of 4 genes involved in EO biosynthesis used for qRT-PCR. [file DataSheet1.docx]

**Supplements**

**Supplement 1. Primers for the expression analysis of 4 genes involved in EO biosynthesis used for qRT-PCR.**

| Accession number | Amplicon size | Tm | GC % | Primers sequence | Primers |
| --- | --- | --- | --- | --- | --- |
| JN701461.1 | 137 | 45.0 | 63.4 | 5’-GTGGGTGAATCTTGTTGAGT-3’ | 1,8-cinoel F |
|  |  | 50.0 | 65.7 | 5’-GGAAGAAGAGGTGGGAAAGT-3’ | 1,8-cinoel R |
| DQ263741.1 | 136 | 42.9 | 64.7 | 5’-TTCAAGCACGACGATACAAAG-3’ | Linaool F |
|  |  | 42.9 | 65.5 | 5’-CACCATCCTCATCAAGTTTTC-3’ | Linalool R |
| HQ404305.1 | 80 | 50.0 | 65.7 | 5’-CAATGAAGCAACTCCCTACC-3’ | β-phellandren F |
|  |  | 52.6 | 63.9 | 5’-AGTATCGTAAGCCACCTCG-3’ | β-phellandren R |
| JX501518.1 | 73 | 45.0 | 61.5 | 5’-GGGATAGGAGAGTTTCAAAG-3’ | α-Pinene F |
|  |  | 47.4 | 58.7 | 5’-TGTAAGGAGCAAGAGAGTC-3’ | α-Pinene R |
| LC373552.1 | 85 | 38.0 | 70.5 | 5’-CGCCAAGGAAAACAAAATGAA-3’ | 18S rRNA F |
|  |  | 50.0 | 64.5 | 5’-ATGACATTCGATAGACGCC-3’ | 18S rRNA R |

**Supplement 2. Components used for gene expression analysis, using qRT-PCR**

| (µl) | concentration | Components |
| --- | --- | --- |
| 0.5 | 200 ng/μl | cDNA |
| 0.5 | 20 P mol | Forward Primer |
| 0.5 | 20 P mol | Reverse Primer |
| 5.0 | 2 X | PCR Master Mix |
| 3.5 | ------------- | Deionized H_2_O |
| 10 |  | Total volume |

**Supplement 3. The ANOVA on the effect of treatment (stress levels) on total protein and chlorophyll contents in lavender plants under drought stress (20 %, 40 %, 60 % and 80 % field capacity) revealed by qRT-PCR**

| MS | | | | | |
| --- | --- | --- | --- | --- | --- |
| S.V | df | Total protein | Chlorophyll a | Chlorophyll b | Total chlorophyll |
| Treatment | 3 | 24.04** | 4.27** | 29.28** | 55.36** |
| Error | 8 | 0.17 | 0.15 | 0.27 | 0.17 |

**Supplement 4. The ANOVA on the EO content in lavender plants under different levels of drought stress (20 %, 40 %, 60 % and 80 % field capacity).**

| Source | df | SS | MS |
| --- | --- | --- | --- |
| EO (ml) | 3 | 0.51 | 0.17** |
| Error | 8 | 0.01 | 0.001 |
| Total | 11 | 0.53 |  |

**** indicate significant at 1% probability level**

**Spplement 5. Comparison between the components and their quantities in this study with the findings reported by Hassanpouraghdam et al. (2011).**

| **Compound** | **80%** | **60%** | **40%** | **20%** | **Hassanpouraghdam et al. (2011)** |
| --- | --- | --- | --- | --- | --- |
| **α- Pinene** | 1.566 | 2.126 | 2.893 | 2.405 | 1.6 |
| **Camphene** | 0.939 | 0.944 | 1.227 | 1.085 | 1.1 |
| **β- Pinene** | 1.767 | 2.956 | 3.759 | 3.333 | 1.4 |
| **β- Myrcene** | 0.479 | 0.808 | 1.192 | 0.946 | ----- |
| **Delta-3- Carene** | 0.912 | 1.408 | 1.925 | 1.474 | ----- |
| **1,8- Cineole** | 41.281 | 40.336 | 36.640 | 38.394 | 31.9 |
| **β- Ocimene y** | 0.081 | 0.084 | 0.136 | 0.080 | ----- |
| **γ- Terpinene** | 0.107 | 0.105 | 0.137 | 0.108 | 0.4 |
| **Camphenilone** | 0.159 | 0.108 | 0.122 | 0.143 | 1.3 |
| **Camphor** | 18.770 | 14.461 | 17.905 | 14.344 | 16.1 |
| **Pinocarvone** | 0.367 | 0.607 | 0.569 | 0.644 | 0.9 |
| **Borneol** | 17.885 | 18.058 | 15.481 | 20.130 | 24.0 |
| **Cryptone** | 2.217 | 2.307 | 2.545 | 2.185 | 3.5 |
| **P-Cymen-8-ol** | 0.962 | 0.699 | 1.261 | 1.124 | 0.5 |
| **Terpineol** | 1.296 | 2.225 | 1.813 | 1.827 | 1.1 |
| **Myrtenol** | 0.359 | 0.633 | 0.547 | 0.489 | 1.3 |
| **1-Verbenone** | 0.555 | 0.708 | 0.738 | 0.728 | ----- |
| **Borneol- acetate** | 2.995 | 2.067 | 1.431 | 1.912 | ----- |
| **Cuminic Aldehyde** | 1.747 | 1.656 | 1.672 | 1.604 | 2.2 |
| **Carvone** | 0.697 | 0.787 | 0.845 | 0.841 | 0.9 |
| **Piperitone** | 0.329 | 0.279 | 0.334 | 0.275 | 0.3 |
| **P-Cymen-7-ol** | 0.643 | 0.731 | 0.817 | 0.669 | 0.1 |
| **Allyl Tiglate** | 0.394 | 0.543 | 0.501 | 0.463 | 0.1 |
| **β- Caryophyllene** | 0.425 | 0.502 | 0.71 | 0.519 | 1.3 |
| **α-Amorphene** | 0.784 | 0.961 | 1.165 | 0.973 | ----- |
| **α- Cadinol** | 2.063 | 3.658 | 3.368 | 3.138 | 1.1 |
| **β- Gurjunene** | 0.216 | 0.244 | 0.221 | 0.168 | ----- |
| **Linalool** | ----- | ----- | ----- | ----- | 0.7 |
| **Sum** | 99.995 | 100.00 | 99.954 | 100.00 | ----- |

**Supplement 6. ANOVA result on the main compositions of essential oils in lavender plants under drought stress** **(20 %, 40 %, 60 % and 80 % field capacity).**

|  | MS | | | | | | | |
| --- | --- | --- | --- | --- | --- | --- | --- | --- |
| Variables | df | EO percentage | α-Pinene | β-Pinene | 1,8-Cineole | Camphor | Borneol | α-Cadinol |
| Treatment | 3 | 0.210** | 0.614** | 1.467** | 8.557** | 10.561** | 7.230** | 0.976** |
| Error | 8 | 0.008 | 0.002 | 0.006 | 1.246 | 9.096 | 0.237 | 0.004 |
| Total | 11 |  |  |  |  |  |  |  |

**** indicate significant at 1% probability level**

**Supplement 7. The percentage of monoterpene hydrocarbons, oxygenated sesquiterpenes, sesquiterpene hydrocarbons and oxygenated sesquiterpenes in the EO of lavender plants under different levels of drought stress (20 %, 40 %, 60 % and 80 % field capacity).**

| FC | monoterpene hydrocarbons | oxygenated monoterpenes | sesquiterpene hydrocarbons | oxygenated sesquiterpenes |
| --- | --- | --- | --- | --- |
| 20 | 9.43^b^ | 85.77^c^ | 1.66^c^ | 3.14^c^ |
| 40 | 11.27^a^ | 83.22^d^ | 2.10^a^ | 3.37^b^ |
| 60 | 8.43^c^ | 86.21^b^ | 1.71^b^ | 3.66^a^ |
| 80 | 5.85^d^ | 90.66^a^ | 1.43^d^ | 2.06^d^ |

**Supplement 8. ANOVA result on the antibacterial activity of essential oils of plants under drought stress (20 %, 40 %, 60 % and 80 % field capacity).**

| MS | | |
| --- | --- | --- |
| S.V | df | Antibacterial property |
| Treatment | 5 | 41.02** |
| Bacteria | 3 | 8.92** |
| T × B | 15 | 4.88** |
| Error | 48 | 0.65 |

**** indicate significant at 1% probability level**
